# Thomson scattering in inhomogeneous plasmas: The Role of the Fluctuation-Dissipation Theorem

**DOI:** 10.1038/s41598-018-25319-6

**Published:** 2018-05-21

**Authors:** V. V. Belyi

**Affiliations:** IZMIRAN, Russian Academy of Sciences, Troitsk, Moscow, 108840 Russia

## Abstract

A self-consistent kinetic theory of Thomson scattering of an electromagnetic field by a non-uniform plasma is derived. We draw the readers’ attention to the inconsistency in recent results on the Thomson scattering in inhomogeneous plasma, which leads to violation of the Fluctuation-Dissipation Theorem. We show, that not only the imaginary part, but also the derivatives of the real part of the dielectric susceptibility determine the amplitude and the width of the Thomson scattering spectral lines. As a result of inhomogeneity, these properties become asymmetric with respect to inversion of the sign of the frequency. A method is proposed for measuring local gradients of the electron density with the aid of Thomson scattering.

Arising from: P. Kozlowski, *et al*. *Sci. Rep*. **6**, 24283 (2016); 10.1038/srep24283.

## Introduction

When an electromagnetic wave propagates in a plasma, its interaction with fluctuational oscillations of the plasma may result in scattering of the wave, which can be accompanied by a change in its frequency and wave vector. The intensity of scattered waves depends on both the intensity of the incident wave and the level of plasma fluctuations. Since the spectrum of plasma fluctuations exhibits sharp maxima at proper plasma frequencies, the spectrum of scattered waves will also exhibit sharp maxima at frequencies differing from the frequency of the incident wave by the according frequencies of the plasma fluctuations. The shift, width and shape of spectral lines carry information on such parameters of the plasma as its density, temperature, mean velocity, ion composition etc. A method of remote probing of a plasma, termed Thomson scattering, is a powerful plasma diagnostic tool that is widely employed in measurements of plasma parameters over a fairly broad range of plasma densities from the ionosphere to strongly coupled plasma. In such measurements the plasma must be transparent to the probe electromagnetic radiation. This may be microwave^1^, laser^2^ or X-ray radiation. A comprehensive exposition of the state-of-the-art of X -ray Thomson scattering is presented in Review^3^.

The differential Thomson scattering cross section, within an elementary solid angle $d\theta ^{\prime}$ and for a frequency interval $d\omega ^{\prime}$ is described by the expression^4,5^:

1 $$d{\rm{\Xi }}=\frac{1}{4\pi }{(\frac{{e}^{2}}{{m}_{e}{c}^{2}})}^{2}\frac{{\omega ^{\prime} }^{2}}{{\omega }_{0}^{2}}\sqrt{\frac{\varepsilon (\omega ^{\prime} )}{\varepsilon ({\omega }_{0})}}(1+{\cos }^{2}\theta ){(\delta {n}_{e}\delta {n}_{e})}_{{\bf{k}}{\omega }}d\theta ^{\prime} d\omega ^{\prime} ,$$

where ${\bf{k}}={\bf{k}}{\boldsymbol{^{\prime} }}-{{\bf{k}}}_{{\bf{0}}}$, $\omega =\omega ^{\prime} -{\omega }_{0};\,{{\bf{k}}}_{{\bf{0}}},\,{\bf{k}}{\boldsymbol{^{\prime} }},\,{\omega }_{0},\,\omega ^{\prime}$ are the wave vectors and the frequencies of the incident and scattered electromagnetic fields. Thus, the problem reduces to finding the spectral characteristics of electron density fluctuations ${(\delta {n}_{e}\delta {n}_{e})}_{\omega ,{\bf{k}}}=S({\bf{k}},\omega )$ - the dynamic electron structure factor. The theory of equilibrium and nonequilibrium plasma fluctuations was successfully developed in the second half of the past century^5–9^. In accordance with the Poisson equation, the dynamic electron structure factor in a spatially homogeneous system is directly linked to the electrostatic field fluctuations. In thermodynamic equilibrium, the electrostatic field fluctuations satisfy the famous Callen-Welton Fluctuation-Dissipation Theorem (FDT)^10^, linking their intensity to the *imaginary* part of the dielectric function *ε*(*ω*, **k**) and to the temperature T

2 $${(\delta {\bf{E}}\delta {\bf{E}})}_{\omega ,{\bf{k}}}=\frac{8\pi \,T{\rm{Im}}\varepsilon (\omega ,{\bf{k}})}{\omega {|\varepsilon (\omega ,{\bf{k}})|}^{2}}.$$

Eq. (2) refers to the steady state, for a space uniform system. However, it is not evident that the plasma parameters can be kept *constant* in both space and time. Inhomogeneities in space and time of these quantities will certainly also contribute to the fluctuations. Hence it is challenging to formulate the generalization of the FDT for inhomogeneous plasma and reformulate accordingly the results for the Thomson scattering.

An attempt to solve this important problem of describing Thomson scattering in an inhomogeneous plasma has been made recently in^11^. The authors proposed the following *a*d hoc generalization for the quantity *S*(**k**, *ω*):

3 $$S({\bf{k}},\omega )=\frac{S{({\bf{k}},\omega )}^{id}}{{|\varepsilon ({\bf{k}},\omega )|}^{2}},$$

“where $S{({\bf{k}},\omega )}^{id}$ is the dynamic structure factor for an ideal (noniteracting gas), and the dielectric (screening) function *ε*(**k**, *ω*) in the denominator of Eq. (3) in a first order gradient expansion in microscopic variable is:

4 $$\varepsilon ({\bf{k}},\omega )=1+\chi ({\bf{k}},\omega )=1+(1+i\frac{\partial }{\partial \omega }\frac{\partial }{\partial t}-i\frac{\partial }{\partial {\bf{r}}}\cdot \frac{\partial }{\partial {\bf{k}}}){\chi }^{eq}({\bf{k}},\omega ),$$

*χ*^*eq*^ is the susceptibility of the ideal Coulomb plasma. The index “eq” labels the susceptibility for a homogeneous system in thermodynamic equilibrium”^11^. Noteworthily, while the authors applied the expansion for the denominator, the numerator in Eq. (3) has not been correspondingly expanded. Although, this approximation based on the “physical intuition” reflects some properties of the system, it fails, unfortunately, to satisfy the basic principles. This entails a dramatic inaccuracy of this approach. Namely, this resulted in two consequences: Firstly, the obtained result is nonphysical, since it contradicts FDT in the local equilibrium state. The FDT for a local equilibrium state was proved by Balescu^12^. The parameters of a system in a local equilibrium state can be changed adiabatically on a scale greater than the particle mean free path. Inhomogeneity and nonstationarity of plasma fluctuations are manifested via a nonlocal dependence upon time^13^ and coordinates^14^. The FDT for a nonlocal plasma was given in our paper^15^. Secondly, the obtained correction due to the inhomogeneity in the denominator Eq. (3) is erroneous for Langmuir oscillations, especially in the case of small wave numbers *k* < *k*_*D*_, which usually occurs in experiments. And last but not least: the rigorous kinetic theory predicts asymmetry of spectral lines in an inhomogeneous plasma. Such asymmetry has been indeed detected in spectroscopic studies of plasma flows in magnetic traps^16,17^.

In the present paper, applying the Klimontovich-Langevin approach^18^ and the time-space multiscale technique, we show that not only the *imaginary* part but also the derivatives of the *real* part of the dielectric susceptibility determine the amplitude and width of spectral lines of the electrostatic field fluctuations and of the dynamic electron structure factor, as well. As a result of the inhomogeneity, these properties become asymmetric with respect to inversion of the sign of the frequency. In the kinetic regime the structure factor is more sensitive to space gradients than the spectral function of the electrostatic field fluctuations. Note that for simple fluids and gases a general theory of hydrodynamic fluctuations for nonequilibrium stationary inhomogeneous states has been developed in^19,20^. In particular, it has been found that there exists an asymmetry of the spectrum for Brillouin scattering from a fluid in a shear flow or in a temperature gradient. The situation for the plasma problem we are considering is, however, quite different.

## Results

To treat the problem, a kinetic approach is required, especially when the wavelength of the fluctuations is larger than the Debye wavelength. To derive nonlocal expressions for the spectral function of the electrostatic field fluctuation and for the dynamic electron structure factor, we adopt the Klimontovich-Langevin approach to describe kinetic fluctuations^18^. A kinetic equation for the fluctuation *δ f*_*a*_ of the one-particle distribution function (DF) with respect to the reference state *f*_*a*_ is considered. In the general case the reference state is a nonequilibrium DF which varies in space and time both on the kinetic scale (mean free path *l*_*ei*_ and interparticle collision time ${{\nu }_{ei}}^{-1}$) and, also, on the larger hydrodynamic scales. These scales are much larger than the characteristic fluctuation time *ω*^−1^. In the nonequilibrium case we can, therefore, introduce a small parameter $\mu ={\nu }_{ei}/\omega$, which allows us to describe fluctuations on the basis of a multiple space and time scale analysis. Obviously, the fluctuations vary on both the “ fast” (**r**, *t*) and the “slow” (*μ***r**, *μt*) space and time scales: $\delta {f}_{a}({\bf{x}}{\boldsymbol{,}}t)=\delta {f}_{a}({\bf{x}},t,\mu {\bf{r}},\mu t)$ and ${f}_{a}({\bf{x}},t)={f}_{a}({\bf{p}},\mu {\bf{r}},\mu t\mathrm{)}.$ Here **x** stands for the phase-space coordinates (**r**, **p**). The Langevin kinetic equation for $\delta {f}_{a}$ has the form^18^

5 $${\widehat{L}}_{a{\bf{x}}t}(\delta {f}_{a}({\bf{x}},t)-\delta {f}_{a}^{S}({\bf{x}},t))=-\,{e}_{a}\delta {\bf{E}}{\boldsymbol{(}}{\bf{r}},t{\boldsymbol{)}}\cdot \frac{\partial {f}_{a}({\bf{x}}{\boldsymbol{,}}t)}{\partial {\bf{p}}},$$

where *e*_*a*_ is the charge of the particle of species *a*, *δ***E** is the electrostatic field fluctuation, and the operator ${\widehat{L}}_{a{\bf{x}}t}$ is defined by

6 $${\widehat{L}}_{a{\bf{x}}t}=\frac{\partial }{\partial t}+{\bf{v}}\cdot \frac{\partial }{\partial {\bf{r}}}+{\widehat{{\rm{\Gamma }}}}_{a}({\bf{x}},t);\,\,{\widehat{{\rm{\Gamma }}}}_{a}({\bf{x}},t)={e}_{a}{\bf{E}}\cdot \frac{\partial }{\partial {\bf{p}}}-\delta {\widehat{I}}_{a},$$

and $\delta {\widehat{I}}_{a}$ is the linearized collision operator. A model collision operator for plasma is presented in^21^.

The Langevin source $\delta {f}_{a}^{S}$ in Eq. (5) is determined by the following equation:

7 $${\widehat{{L}}}_{a{\bf{x}}t}{\overline{\delta {f}_{a}({\bf{x}},t)\delta {f}_{b}({\bf{x}}{\boldsymbol{^{\prime} }},t^{\prime} )}}^{S}={\delta }_{ab}\delta (t-t^{\prime} )\delta ({\bf{x}}-{\bf{x}}{\boldsymbol{^{\prime} }}){f}_{b}({\bf{x}}{\boldsymbol{^{\prime} }},t^{\prime} ).$$

The solution of Eq. (5) has the form

8 $$\delta {f}_{a}({\bf{x}},t)=\delta {f}_{a}^{S}({\bf{x}},t)-\sum _{b}\int d{\bf{x}}{\boldsymbol{^{\prime} }}{\int }_{-\infty }^{t}dt^{\prime} {G}_{ab}({\bf{x}},t,{\bf{x}}{\boldsymbol{^{\prime} }},t^{\prime} ){e}_{b}\delta {\bf{E}}({\bf{r}}{\boldsymbol{^{\prime} }},t^{\prime} )\cdot \frac{\partial {f}_{b}({\bf{x}}{\boldsymbol{^{\prime} }},t^{\prime} )}{\partial {\bf{p}}{\boldsymbol{^{\prime} }}},$$

where the Green function ${G}_{ab}({\bf{x}},t,{\bf{x}}{\boldsymbol{^{\prime} }},t^{\prime} )$ of the operator ${\widehat{L}}_{a{\bf{x}}t}$ is determined by

9 $${\widehat{{L}}}_{a{\bf{x}}t}{G}_{ab}({\bf{x}},t,{\bf{x}}{\boldsymbol{^{\prime} }},t^{\prime} )={\delta }_{ab}\delta ({\bf{x}}-{\bf{x}}{\boldsymbol{^{\prime} }})\delta (t-t^{\prime} )$$

with the causality condition:

10 $${G}_{ab}({\bf{x}},t,{\bf{x}}{\boldsymbol{^{\prime} }},t^{\prime} )=\mathrm{0,}$$

when $$t < t^{\prime} $$.

Thus, ${\overline{\delta {f}_{a}({\bf{x}},t)\delta {f}_{b}({\bf{x}}{\boldsymbol{^{\prime} }},t^{\prime} )}}^{S}$ and ${G}_{ab}({\bf{x}},t,{\bf{x}}{\boldsymbol{^{\prime} }},t^{\prime} )$ are connected by the relation:

11 $${\overline{\delta {f}_{a}({\bf{x}},t)\delta {f}_{b}({\bf{x}}{\boldsymbol{^{\prime} }},t^{\prime} )}}^{S}={G}_{ab}({\bf{x}},t,{\bf{x}}{\boldsymbol{^{\prime} }},t^{\prime} ){f}_{b}({\bf{x}}{\boldsymbol{^{\prime} }},t^{\prime} ),\,t > t^{\prime} .$$

For stationary and spatially uniform systems the DF *f*_*a*_ and the operator ${\widehat{{\rm{\Gamma }}}}_{a}$ do not depend on time and space. In this case, the dependence on time and space of the Green function ${G}_{ab}({\bf{x}},t,{\bf{x}}{\boldsymbol{^{\prime} }},t^{\prime} )$ is manifested only through the difference $t-t^{\prime}$ and ${\bf{r}}-{\bf{r}}{\boldsymbol{^{\prime} }}$. However, when the DF ${f}_{a}({\bf{p}}{\boldsymbol{,}}\mu {\bf{r}}{\boldsymbol{,}}\mu t)$ and ${\widehat{{\rm{\Gamma }}}}_{a}({\bf{p}}{\boldsymbol{,}}\mu {\bf{r}}{\boldsymbol{,}}\mu t)$ are slowly varying quantities in time and space, and when nonlocal effects are considered, the time and space dependence of ${G}_{ab}({\bf{x}},t,{\bf{x}}{\boldsymbol{^{\prime} }},t^{\prime} )$ is more subtle:

12 $${G}_{ab}({\bf{x}},t,{\bf{x}}{\boldsymbol{^{\prime} }},t^{\prime} )={G}_{ab}({\bf{p}},{\bf{p}}{\boldsymbol{^{\prime} }},{\bf{r}}-{\bf{r}}{\boldsymbol{^{\prime} }},t-t^{\prime} ,\mu {\bf{r}}{\boldsymbol{^{\prime} }},\mu t^{\prime} ).$$

For the homogeneous case this non-trivial result was obtained for the first time in our previous work^13^. This result was extended to inhomogeneous systems^14^. Here we want to stress that the nonlocal effects appear due to the slow time and space dependencies $\mu {\bf{r}}{\boldsymbol{^{\prime} }}$ and $\mu t^{\prime}$.

Relationship (12) is directly linked with the constitutive relation between the electric displacement and the electric field

13 $${D}_{i}({\bf{r}},t)=\int d{\bf{r}}{\boldsymbol{^{\prime} }}{\int }_{-\infty }^{t}dt^{\prime} {\varepsilon }_{ij}({\bf{r}},{\bf{r}}{\boldsymbol{^{\prime} }},t,t^{\prime} ){E}_{j}({\bf{r}}{\boldsymbol{^{\prime} }},t^{\prime} ).$$

Previously two kinds of constitutive relations were proposed phenomenologically for a weakly inhomogeneous and slowly time-varying medium. Kadomtsev^22^ formulated the so-called *symmetrized* constitutive relation.

14 $${D}_{i}({\bf{r}},t)=\int d{\bf{r}}{\boldsymbol{^{\prime} }}{\int }_{-\infty }^{t}dt^{\prime} {\varepsilon }_{ij}({\bf{r}}-{\bf{r}}{\boldsymbol{^{\prime} }},t-t^{\prime} ,\mu \frac{{\bf{r}}+{\bf{r}}{\boldsymbol{^{\prime} }}}{2},\mu \frac{t+t^{\prime} }{2}){E}_{j}({\bf{r}}{\boldsymbol{^{\prime} }},t^{\prime} ).$$

Rukhadze and Silin^23^ proposed a *nonsymmetrized* constitutive relation

15 $${D}_{i}({\bf{r}},t)=\int d{\bf{r}}{\boldsymbol{^{\prime} }}{\int }_{-\infty }^{t}dt^{\prime} {\varepsilon }_{ij}({\bf{r}}-{\bf{r}}{\boldsymbol{^{\prime} }},t-t^{\prime} ,\mu {\bf{r}},\mu t){E}_{j}({\bf{r}}{\boldsymbol{^{\prime} }},t^{\prime} ).$$

Both phenomenological formulations are unsatisfactory. The correct expression should be

16 $${D}_{i}({\bf{r}},{t})=\int d{\bf{r}}{\boldsymbol{^{\prime} }}{\int }_{-\infty }^{t}dt^{\prime} {\varepsilon }_{ij}({\bf{r}}-{\bf{r}}{\boldsymbol{^{\prime} }},t-t^{\prime} ,\mu {\bf{r}}{\boldsymbol{^{\prime} }},\mu t^{\prime} ){E}_{j}({\bf{r}}{\boldsymbol{^{\prime} }},t^{\prime} ).$$

In the first order, expansion with respect to *μ*, Eq. (8) leads to

17 $$\begin{array}{rcl}\delta {f}_{a}({\bf{x}},t) & = & \delta {f}_{a}^{S}({\bf{x}},t)-\sum _{b}{e}_{b}\int d{\bf{p}}{\boldsymbol{^{\prime} }}d{\rho }{\int }_{0}^{\infty }d\tau (1-\mu \tau \frac{\partial }{\partial \mu t}-\mu {\rho }\cdot \frac{\partial }{\partial \mu {\bf{r}}})\\  &  & \times {G}_{ab}({\rho },\tau ,{\bf{p}}{\boldsymbol{,}}{\bf{p}}{\boldsymbol{^{\prime} }},\mu {\bf{r}},\mu t)\delta {\bf{E}}({\bf{r}}-{\rho },t-\tau )\cdot \frac{\partial {f}_{b}({\bf{p}}{\boldsymbol{^{\prime} }},\mu {\bf{r}},\mu t)}{\partial {\bf{p}}{\boldsymbol{^{\prime} }}},\end{array}$$

with $${\rho }={\bf{r}}-{\bf{r}}{\boldsymbol{^{\prime} }}$$ and $$\tau =t-t^{\prime} $$.

Using the Poisson equation and performing the Fourier-Laplace transformation for the fast variables

18 $$\delta {\bf{E}}({\bf{k}},\omega )={\int }_{0}^{\infty }dt\int d{\bf{r}}\delta {\bf{E}}({\bf{r}},t)\exp (\,-\,{\rm{\Delta }}t+i\omega t-i{\bf{k}}\cdot {\bf{r}}),$$

we have that the spectral function of the nonequilibrium electrostatic field fluctuations assumes the form:

19 $${(\delta {\bf{E}}\delta {\bf{E}})}_{\omega ,{\bf{k}}}=\frac{32{\pi }^{2}}{{|\varepsilon (\omega ,{\bf{k}})|}^{2}}\sum _{a}{e}_{a}^{2}Re\int d{\bf{p}}(1+i\mu \frac{\partial }{\partial \omega }\frac{\partial }{\partial \mu t}-i\mu \frac{\partial }{\partial {\bf{k}}}\cdot \frac{\partial }{\partial \mu {\bf{r}}})\frac{1}{{k}^{2}}{\widehat{L}}_{a\omega {\bf{k}}}^{-1}{f}_{a}({\bf{p}},\mu {\bf{r}},\mu t),$$

where we introduced the effective dielectric function as:

20 $$\varepsilon (\omega ,{\bf{k}})=1+\sum _{a}{\chi }_{a}(\omega ,{\bf{k}});\,{\chi }_{a}(\omega ,{\bf{k}})=(1+i\mu \frac{\partial }{\partial \omega }\frac{\partial }{\partial \mu t}-i\mu \frac{\partial }{\partial \mu {\bf{r}}}\cdot \frac{\partial }{\partial {\bf{k}}}){\chi }_{a}^{Neq}(\omega ,{\bf{k}},\mu {\bf{r}},\mu t),$$

where

21 $${\chi }_{a}^{Neq}(\omega ,{\bf{k}},\mu {\bf{r}},\mu t)=-\,\frac{4\pi i{e}_{a}^{2}}{{k}^{2}}\int d{\bf{p}}{\widehat{L}}_{a\omega {\bf{k}}}^{-1}{\bf{k}}\cdot \frac{\partial }{\partial {\bf{p}}}{f}_{a}({\bf{p}}{\boldsymbol{,}}\mu {\bf{r}},\mu t)$$

is the susceptibility for a nonequilibrium plasma.

If the authors of paper^11^ deal with the nonequilibrium case, they should use an expression similar to Eq. (19) in Eq. (3) as well as the nonequilibrium susceptibility (21).

For the local equilibrium case, where the reference state ${f}_{a}^{Leq}$ is Maxwellian, we have the identity:

22 $$\begin{array}{c}\int d{\bf{p}}(1+i\mu \frac{\partial }{\partial \omega }\frac{\partial }{\partial \mu t}-i\mu \frac{\partial }{\partial {\bf{k}}}\cdot \frac{\partial }{\partial \mu {\bf{r}}})\frac{1}{{k}^{2}}{\widehat{L}}_{a\omega {\bf{k}}}^{-1}{f}_{a}^{Leq}({\bf{p}},\mu {\bf{r}},\mu t)\\ \,=\,\frac{i}{{\omega }_{a}}\int d{\bf{p}}{f}_{a}^{Leq}({\bf{p}},\mu {\bf{r}},\mu t)\\ \,\,-\,\frac{i{T}_{a}}{4\pi {e}_{a}^{2}{\omega }_{a}}(1+i\mu \frac{\partial }{\partial \omega }\frac{\partial }{\partial \mu t}-i\mu \frac{\partial }{\partial \mu {\bf{r}}}\cdot \frac{\partial }{\partial {\bf{k}}}){\chi }_{a}^{Leq}(\omega ,{\bf{k}},\mu {\bf{r}},\mu t),\end{array}$$

and Eq. (19) takes the form

23 $${(\delta {\bf{E}}\delta {\bf{E}})}_{\omega ,{\bf{k}}}=\sum _{a}\frac{8\pi \,{T}_{a}}{{\omega }_{a}{|\varepsilon {\boldsymbol{(}}\omega ,{\bf{k}})|}^{2}}Im{\chi }_{a}(\omega ,{\bf{k}}),$$

where ${\omega }_{a}=\omega -{\bf{k}}{{\bf{V}}}_{a}$. For the case of equal temperatures and **V**_a_ = 0, Eq. (23) satisfies the FDT. In this case the small parameter *μ* is determined on the slower hydrodynamic scale. Imaginary part of the susceptibility $\chi ({\bf{k}},\omega )$ determines the width of the spectral line ${(\delta {\bf{E}}\delta {\bf{E}})}_{\omega ,{\bf{k}}}$ near the resonance:

24 $$\gamma =(Im{\chi }^{Leq}+\mu \frac{\partial }{\partial \omega }\frac{\partial }{\partial \mu t}Re{\chi }^{Leq}-\mu \frac{\partial }{\partial \mu {\bf{r}}}\cdot \frac{\partial }{\partial {\bf{k}}}Re{\chi }^{Leq})/\frac{\partial }{\partial \omega }Re{\chi }^{Leq}.$$

In Eq. (24) there appear additional first-order terms of the small parameter *μ*. It is important to note that the *imaginary* part of the dielectric susceptibility is now replaced by the *real* part, which in the plasma resonance may be greater than the *imaginary* part by the same factor *μ*^−1^. Therefore, the second and third terms in Eq. (24) in the kinetic regime have an effect comparable to that of the first term. Second-order corrections in the expansion in *μ* only appear in the *imaginary* part of the susceptibility, and they can be reasonably neglected. It is therefore sufficient to retain the first-order corrections to resolve the problem. The width of the spectral lines Eq. (24) is affected by new nonlocal terms. They are not related to Joule dissipation and appear because of an additional phase shift between the induction vector and the electric field. This phase shift results from the finite time needed to set the polarization in the plasma with dispersion^24^. Such a phase shift in the plasma with space dispersion appears due to the medium inhomogeneity.

For the case where the system parameters are homogeneous in space but vary in time, the correction to the width of the spectral lines in Eq. (24) is still symmetric with respect to the change in sign of *ω*. However, when the plasma parameters are space dependent this symmetry is lost. The real part of the susceptibility ${\chi }^{Leq}({\bf{k}},\omega )$ in Eq. (24) is an even function of *ω*. This property implies that the contribution of the space derivative to the expression for the width of the spectral lines is odd function of *ω*. Moreover, this term gives rise to an anisotropy in **k** space.

Let us estimate this correction for the plasma mode $(\omega ={\omega }_{L})$

25 $$Re\varepsilon =1-\frac{{\omega }_{L}^{2}}{{\omega }^{2}}(1+3\frac{{k}^{2}T}{m{\omega }^{2}});\,Im\varepsilon =\frac{{\nu }_{ei}}{\omega }$$

and

26 $$\gamma =[{\nu }_{ei}+\frac{2}{n}\frac{\partial n}{\partial t}+6\frac{{\omega }_{L}}{n{k}_{D}^{2}}{\bf{k}}\cdot \frac{\partial n}{\partial {\bf{r}}}sgn\omega ]\mathrm{/2}.$$

On the hydrodynamic scale $|\frac{2}{n}\frac{\partial n}{\partial t}|,\,|{\bf{k}}\cdot \frac{\partial n}{\partial {\bf{r}}}6{\omega }_{L}/n{k}_{D}^{2}| < {\nu }_{ei}$, and *γ* > 0.

For the spatially homogeneous case there is no difference between the spectral properties of the longitudinal electric field and of the electron density, because they are related by the Poisson equation. This statement is no longer valid when an inhomogeneous plasma is considered. Indeed the longitudinal electric field is linked to the particle density by the nonlocal relation:

27 $$\delta {\bf{E}}({\bf{r}},t)=-\,\frac{\partial }{\partial {\bf{r}}}\sum _{a}{e}_{a}\int \frac{1}{|{\bf{r}}-{\bf{r}}{\boldsymbol{^{\prime} }}|}\delta {n}_{a}({\bf{r}}{\boldsymbol{^{\prime} }},t)d{\bf{r}}{\boldsymbol{^{\prime} }}.$$

In the same approximation as in Eq. (23) the expression for the dynamic electron structure factor for a two-component $(a=e,i)$ local equilibrium plasma has the form^15^:

28 $${S}^{e}({\bf{K}},\omega )=\frac{2{n}_{e}{k}^{2}}{{\omega }_{e}{k}_{D}^{2}}{|\frac{1+{\mathop{\chi }\limits^{ \sim }}_{i}({\bf{k}},\omega )}{\mathop{\varepsilon }\limits^{ \sim }({\bf{k}},\omega )}|}^{2}{\rm{I}}{\rm{m}}\,{\mathop{\chi }\limits^{ \sim }}_{e}({\bf{k}},\omega )+{|\frac{{\mathop{\chi }\limits^{ \sim }}_{e}({\bf{k}},\omega )}{\mathop{\varepsilon }\limits^{ \sim }({\bf{k}},\omega )}|}^{2}\frac{{T}_{i}}{{T}_{e}}\frac{2{n}_{e}{k}^{2}}{{\omega }_{i}{k}_{D}^{2}}{\rm{I}}{\rm{m}}\,{\mathop{\chi }\limits^{ \sim }}_{i}({\bf{k}},\omega ),$$

where *k*_*D*_ is the inverse Debye length,

29 $$\tilde{\varepsilon }({\bf{k}},\omega )=1+\sum _{a}{\tilde{\chi }}_{a}({\bf{k}},\omega ),$$

30 $${\tilde{\chi }}_{a}(\omega ,{\bf{k}})=(1+i\mu \frac{\partial }{\partial \omega }\frac{\partial }{\partial \mu t}-i\mu \frac{1}{{k}^{2}}\frac{\partial }{\partial \mu {r}_{i}}{k}_{j}\frac{\partial }{\partial {k}_{i}}{k}_{j}){\chi }_{a}^{Leq}(\omega ,{\bf{k}},\mu {\bf{r}},\mu t\mathrm{)}.$$

The inhomogeneous correction in Eq. (30) $(\frac{1}{{k}^{2}}\frac{\partial }{\partial \mu {r}_{i}}{k}_{j}\frac{\partial }{\partial {k}_{i}}{k}_{j}Re{\chi }_{a}^{Leq})$ is not the same as in Eq. (24) $(\frac{\partial }{\partial \mu {\bf{r}}}\cdot \frac{\partial }{\partial {\bf{k}}}Re{\chi }^{Leq})$. The origin of this difference is that the Green functions for the electrostatic field fluctuations and particle density fluctuations are not the same in an inhomogeneous situation. As above, we can expand $\tilde{\varepsilon }(\omega ,{\bf{k}})$ near the plasma resonance $\omega ={\omega }_{L}$. Thus, for the Langmuir line

31 $${S}^{e}({\bf{k}},\omega )=\frac{\tilde{\gamma }}{{(\omega -{\omega }_{L}sgn\omega )}^{2}+{\tilde{\gamma }}^{2}}\frac{2{n}_{e}{k}^{2}}{\omega {k}_{D}^{2}\partial Re\varepsilon /\partial \omega }{\lfloor }_{\omega ={\omega }_{L}},$$

where

32 $$\tilde{\gamma }=[Im\varepsilon +\mu \frac{{\partial }^{2}Re\varepsilon }{\partial \mu t\partial \omega }-\mu \frac{1}{{k}^{2}}\frac{\partial }{\partial \mu {r}_{i}}{k}_{j}\frac{\partial }{\partial {k}_{i}}{k}_{j}Re\varepsilon ]/\frac{\partial Re\varepsilon }{\partial \omega }{\lfloor }_{\omega ={\omega }_{L}sgn\omega }$$

is the width of the dynamic electron structure factor. An estimate for the plasma mode is then:

33 $$\tilde{\gamma }=[{\nu }_{ei}+\frac{2}{n}\frac{\partial n}{\partial t}+\frac{{\omega }_{L}}{n{k}^{2}}{\bf{k}}\cdot \frac{\partial n}{\partial {\bf{r}}}(1+\frac{9{k}^{2}}{{k}_{D}^{2}})sgn\omega ]\mathrm{/2}.$$

**Figure 1:**
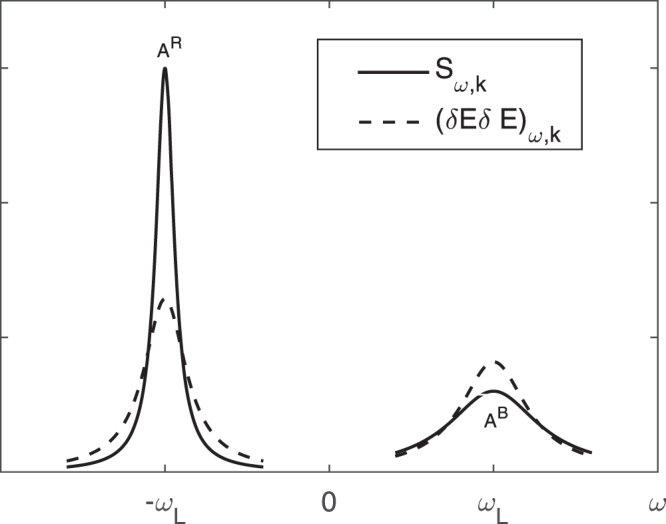
The dynamic electron structure factor ${S}^{e}({\bf{k}},\omega )$ (solid line) and the spectral function of electrostatic field fluctuations ${(\delta {\bf{E}}\delta {\bf{E}})}_{\omega ,{\bf{k}}}$ (dashed line) as a function of frequency. ${k}_{D}/k=3$; ${\bf{k}}\cdot \partial n/n\partial {\bf{r}}={\nu }_{ei}{k}_{D}\mathrm{/27}{v}_{T}$.

From this equation we see that the inhomogeneous correction in Eq. (33) is greater than the one in Eq. (26) by the factor $(1+{k}_{D}^{2}\mathrm{/9}{k}^{2})\mathrm{3/2}$. For the same inhomogeneity, i.e., the same gradient of the density, we plot the dynamic electron structure factor ${S}^{e}({\bf{k}},\omega )$ together with the ${(\delta {\bf{E}}\delta {\bf{E}})}_{\omega ,{\bf{k}}}$ as functions of frequency (Fig. 1). This figure shows that the asymmetry of the spectral lines is present both for ${S}^{e}({\bf{k}},\omega )$ and ${(\delta {\bf{E}}\delta {\bf{E}})}_{\omega ,{\bf{k}}}$. However, this effect is more pronounced in ${S}^{e}({\bf{k}},\omega )$ than in ${(\delta {\bf{E}}\delta {\bf{E}})}_{\omega ,{\bf{k}}}$. Such asymmetry has been indeed detected in inhomogeneous plasma^16,17^. The asymmetry of lines ${S}^{e}({\bf{k}},\omega )$ can be used as a new diagnostic tool for measuring local gradients in plasmas by Thomson scattering.

The Langmuir line (31) takes the Lorentz form. The amplitude of the spectral line *A* is inversely proportional to its width

34 $$A=\frac{{n}_{e}{k}^{2}}{\tilde{\gamma }{k}_{D}^{2}}.$$

From Eq. (34) and Eq. (33) quite a simple formula for calculation of the electron density gradient from the Thomson scattering spectrum follows:

35 $${\bf{k}}\cdot \frac{\partial n}{n\partial {\bf{r}}}=\frac{{\nu }_{ei}}{{v}_{T}}\frac{{A}^{R}-{A}^{B}}{{A}^{R}+{A}^{B}}\frac{{k}_{D}}{{k}_{D}^{2}/{k}^{2}+9}=\frac{({\gamma }^{R}+{\gamma }^{B})}{{\omega }_{L}}\frac{{A}^{R}-{A}^{B}}{{A}^{R}+{A}^{B}}\frac{{k}_{D}^{2}}{{k}_{D}^{2}/{k}^{2}+9},$$

here ${A}^{R},{A}^{B}$ and ${\gamma }^{R},{\gamma }^{B}$ are the amplitudes and the half-widths of the red and blue Langmuir satellites, respectively (Fig. 1).

Thus, intensity and width measurements of the red and blue lines of the spectrum allow to determine the scalar product of the electron density gradient and the scattering vector at a given point. To determine the vector $\partial n/n\partial {\bf{r}}$ it is sufficient to measure the radiation scattered in three directions simultaneously.

Similar calculations can also be performed for degenerate high density plasma^3^.

## Conclusion

A first-principle kinetic theory of Thomson scattering in a non-uniform plasma is constructed, which agrees with the basic FDT and provides quantitatively correct results, that have been confirmed experimentally^16,17^. Moreover, our theory provides a novel and unique method for the remote probing and measurement of electron density gradients in a plasma; this is based on the demonstrated asymmetry of the Thomson scattering lines. The latter may be important for numerous technological applications, e.g. for the tokamak^25^, for the high energy density plasma^3^ etc. Our findings are in a sharp contrast with the results of the recent publication^11^, where the suggested ad hoc theory did not agree with the local FDT (which proved to hold valid) and led to quantitatively (and qualitatively) incorrect predictions.
